# Targeted Delivery of 5-fluorouracil with Monoclonal Antibody Modified Bovine Serum Albumin Nanoparticles

**Published:** 2015

**Authors:** Ghazal Fadaeian, Seyed Abbas Shojaosadati, Hasan Kouchakzadeh, Fazel Shokri, Masoud Soleimani

**Affiliations:** a *Biotechnology Group, Chemical Engineering Faculty, Tarbiat Modares University, Tehran, Iran.*; b *Department of Immunology, School of Public Health, Tehran University of Medical Science, Tehran, Iran.*; c *Monoclonal Antibody Research Center, Avicenna Research Institute, Tehran, Iran.*; d *Department of Hematology, Faculty of Medical Science, Tarbiat Modares University, Tehran, Iran.*

**Keywords:** 5-Flourouracil, Bovine serum albumin nanoparticles, 1F2 monoclonal antibody

## Abstract

Herein, 1F2, an anti-HER2 monoclonal antibody (mAb), was covalently coupled to the surface of 5-Fluorouracil (5-FU) loaded bovine serum albumin (BSA) nanoparticles. Concerning two different crosslinkers for conjugation of 1F2, Maleimide-poly (ethylene glycol)-Succinimidyl carbonate (Mal-PEG5000-NHS) was selected due to its higher conjugation efficiency (23 ± 4%) obtained in comparison to N-succinimidyl 3-(2-Pyridyl Dithio) Propionate (SPDP) (8 ± 2%). A slight increase in the average particle size with a negligible prolongation of the 5-FU release was observed after 1F2 coupling. The 1F2-coupled 5-FU-loaded BSA nanoparticles interacted with nearly all HER2 receptors available on the surface of HER2-positive SKBR3 cells. No cellular uptake was observed for HER2-negative MCF7 cells. Physicochemical and biological properties of the mAb-modified nanoparticles did not significantly alter after three months of storage at room temperature. The *in-vitro* cytotoxicity evaluation by MTT assay, demonstrated lower SKBR3 viability (50.7 ± 9 %) after 5 hours contact with 1F2-coupled 5-FU-loaded BSA nanoparticles in comparison with the other control systems due to their cell attachment and internalization after washing. In addition, no significant toxicity was observed on MCF7 cells. This novel system can efficiently be employed for targeted delivery of 5-FU to HER2-positive cancerous cells.

## Introduction

The effective chemotherapeutic anti-cancer drug, 5-Fluorouracil (5-FU), which has been used against colon, breast, ovarian and skin cancers for about 40 years, belongs to the category of chemotherapeutics called antimetabolites (1). In its mechanism of action, when 5-FU incorporates into the cellular metabolism, interferes with the maturation of nuclear RNA, limit cell division, leading cellular death and causing the tumor to shrink (2). 5-FU is frequently administered in an intravenous (IV) injection and orally through feeds and drinking water (2). Chest pain, cardiotoxicity, low blood counts and cognitive impairment are the most serious side effects of 5-FU (3, 4). Through making use of targeted delivery systems, the therapeutic effects of anti-cancer drugs such as 5-FU will improve and their side effects will decrease due to effective targeting of tumor tissues (5-9).

Bovine serum albumin (BSA) nanoparticles are widely used for drug delivery due to their abundance, low cost, and ease of preparation, purification and scale up against other drug delivery systems (10-12). Albumin nanoparticles can be functionalized with drug targeting ligands such as monoclonal antibodies (mAbs) through the available superficial amino and carboxylic groups (10, 13). Therefore, active targeting of therapeutic agents to tumor tissues is achievable, making use of ligand conjugated BSA nanoparticles.

The human epidermal growth factor receptor-2 (HER2) overexpresses with a significant proportion in breast, ovarian and gastric cancers and may serve as a tumor-targeting marker for the treatment of patients with HER2-positive cells (14, 15). Attachment of anti-HER2 mAb-conjugated BSA nanoparticles to the surface of HER2 overexpressing cells allows an effective internalization of the nanoparticles via receptor-mediated endocytosis leading to an efficient treatment (10).

1F2 is a new murine mAb, which is produced with a novel immunizing protocol that specifically recognizes the extracellular domain of HER2 with an affinity constant (K _aff_) of 5 × 10^9^ (16). This mAb seems to be a promising ligand for conjugation to nanoparticles for targeted delivery of anti-cancer drugs to HER2-positive cancerous cells that previously proved their attachment to BT474 cells (17).

Previously, we prepared 5-FU-loaded BSA nanoparticles and optimized their PEGylation conditions (18, 19). In this study, 1F2 mAb was covalently coupled to the surface of BSA nanoparticles to achieve a novel targetable delivery system for 5-FU. In order to evaluate the effect of crosslinker on the conjugation amount of 1F2, two different long and short chain heterobifunctional crosslinkers, maleimide-poly (ethylene glycol)-succinimidyl carbonate (Mal-PEG-NHS) and N-succinimidyl 3-(2-pyridyl dithio) propionate (SPDP) were employed. The conjugation efficiency of 1F2 mAb to 5-FU-loaded BSA nanoparticles through these crosslinkers was measured by enzyme-linked immunosorbent assay (ELISA) and the best of them was selected. Flow cytometery technique was employed to assess the cellular binding of 1F2-modified 5-FU-loaded BSA nanoparticles to HER2-positive SKBR3 and HER2-negative MCF7 cell lines. Nanoparticle formulations were comprehensively characterized and *in-vitro* cumulative release of 5-FU was carefully examined. Short and long-term physicochemical and biological stability of 1F2-coupled 5-FU-loaded BSA nanoparticles were investigated during 72 hours and three months of storage, respectively. Finally, the specificity and cytotoxicity of BSA nanoparticles, free drug, 5-FU-loaded BSA nanparticles, 5-FU-loaded PEGylated BSA nanoparticles and 1F2-coupled BSA nanoparticles evaluated *in-vitro* on SKBR3 and MCF7 cancerous cells and compared with 1F2-coupled 5-FU-loaded BSA nanoparticles.

## Experimental

*Chemical and biological materials*

Bovine serum albumin (BSA, fraction V, minimum 98%), glutaraldehyde 8% aqueous solution, N-succinimidyl 3-(2-Pyridyl Dithio) Propionate (SPDP), 5-fluorouracil, 3, 3’, 5, 5’ tetramethylbenzidine (TMB) substrate and Methylthiazolyldiphenyl-tetrazolium bromide (MTT) were obtained from Sigma (Steinheim, Germany). 2-iminothiolane (2-IT, Traut’s reagent) and 5,5’ -dithio-bis (2-nitro-benzoic acid) (Ellman’s reagent) were purchased from Pierce (IL, USA). Maleimide-poly (ethylene glycol)-Succinimidyl carbonate with the average molecular weight of 5000 Da (Mal-PEG5000-NHS) was obtained from Jen Kem Technology (Texas, USA). Recombinant extracellular part of HER2 was purchased from eBioscience (CA, USA). The production of 1F2 mAb was reported elsewhere (16). Horse-radish peroxidase (HRP)-conjugated sheep anti-mouse Ig and fluorescein isothiocyanate (FITC)-conjugated sheep anti-mouse immunoglobulin were produced in our laboratory. All other reagents were of analytical grade and used as received.

The SKBR3 and MCF7 cell lines were obtained from National Cell Bank of Iran (NCBI, Pasteur Institute of Iran, Tehran, Iran). The SKBR3 cells were grown in DMEM culture media containing 10% fetal bovine serum (FBS) (Gibco Invitrogen, Carlsbad, CA), 100 U/mL penicillin (Gibco Invitrogen), 1000 U/mL leukemia inhibitory factor (LIF) (Millipore, USA), and 1% glutamine (Gibco Invitrogen). The MCF7 cells were cultured in RPMI 1640 medium (Gibco Invitrogen) containing 15% FBS, 10 µg/mL insulin (Exir Co., Boroojerd, Iran), 100 µg/mL streptomycin, and 100 U/mL penicillin. Both cell lines were incubated at 37  C in a humidified atmosphere containing 5% CO_2_.

*Preparation and separation of 5-FU-loaded BSA nanoparticles*

5-FU-loaded BSA nanoparticles were prepared by a well-known desolvation technique as previously described (18, 20). Briefly, 0.2 g BSA in 2.0 mL of 2 mg/mL 5-FU aqueous solution titrated to pH 8.2 and converted to nanoparticles by continuous addition of ethanol by a syringe pump at the rate of 1.0 mL/min under constant stirring (550 rpm). Subsequently, 120 µL of 8% glutaraldehyde aqueous solution was added drop-wise to induce particles cross-linking. The cross-linking process continued overnight keeping the suspension under stirring. The produced nanoparticles were separated from the solution by three times of centrifugation (25,000 g, 20 min), followed by washing and redispersion in 10 mM NaCl pH 8 to the original volume.

*Thiolation of 1F2 mAb*

A crucial step in the attachment process of mAb to nanoparticles is the introduction of free sulfhydryl groups (thiolation) on mAb structure. Primary amino groups of the mAb can react with 2-IT, leading to creation of active groups suitable for conjugation to nanoparticles. Thiolation of 1F2 mAb was performed under an optimized condition (17). In principle, 1F2 solution (500 µL, 1.2 mg/mL) in phosphate buffer saline (PBS, pH 8.0) and containing 5 mM EDTA was incubated with 100-fold molar excess of 2-IT solution for 1 h at 20 ^°^C. Afterward, the thiolated 1F2 was purified by dialysis against 10.0 mL PBS pH 8.0 for 4 hours with 10 times buffer replacement. The amount of thiol groups produced was quantified with Ellman's reagent based on molar absorptivity method as previously described (17). 

*Conjugation of 1F2 mAb to BSA nanoparticles using Mal-PEG5000-NHS*

Thiolated 1F2 mAb was conjugated to 5-FU-loaded BSA nanoparticles through Mal-PEG5000-NHS. PEGylation of BSA nanoparticles was performed under optimum conditions (18). Briefly, 5 mg/mL of 5-FU-loaded BSA nanoparticles in phosphate buffer (pH 7.0) was incubated with a concentration of Mal-PEG5000-NHS equal to 10-fold molar excess of superficial free amino groups for 30 min at 27 °C under constant shaking. Then, PEGylated nanoparticles were separated by centrifugation and redispersed as mentioned before. For 1F2 coupling, 1 mL of the sulfhydryl-reactive 5-FU-loaded nanoparticle suspension was incubated with 50 μL of the thiolated 1F2 for 12 hours at 20 °C under constant stirring (600 rpm). Then, the 1F2-coupled nanoparticles were separated from unreacted 1F2 mAb by centrifugation and their 1F2 content was measured by indirect ELISA to determine the amount of the conjugated 1F2 mAb to drug-loaded nanoparticles.

*Conjugation of 1F2 mAb to BSA nanoparticles using SPDP*

SPDP is a short-chain crosslinker for amine-to-sulfhydryl conjugation. The amine-reactive NHS ester will react with lysine residues to form a stable amide bond. The other end of the spacer arm, pyridyldithiol reactive groups will react with sulfhydryls to form a disulfide bond. In order to investigate the effect of chain length of the crosslinker on conjugation efficiency, thiolated 1F2 mAb was also coupled to 5-FU-loaded BSA nanoparticles by SPDP under optimum conditions obtained after some pre-experiments. 120 μL of SPDP solution (20 mM) was added to 1 mL of the 5-FU-loaded BSA nanoparticle suspension (5 mg/mL). The resulting solution was kept at room temperature under constant stirring for 1 hour and then, SPDP modified nanoparticles were separated by centrifugation followed by redispersion in phosphate buffer pH 7. SPDP modified nanoparticles were incubated with 50 µL of thiolated 1F2 for 12 h at room temperature. The 1F2-coupled 5-FU-loaded BSA nanoparticles were separated from unreacted 1F2 molecules by centrifugation and their 1F2 content was assessed by indirect ELISA.

*Measurement of the level of monoclonal antibody-coupled to drug-loaded nanoparticles by indirect ELISA*

All reactions were carried out in sealed microtiter polystyrene plates (Maxisorp, Nunc, Roskilde, Denmark) in a volume of 50 µL. Plates were washed three times after each incubation with PBS (0.15 M, pH 7.2) containing 0.05% Tween-20 (Sigma, Germany) (PBS-T). Initially the plate was coated with 0.5 µg/mL recombinant extracellular part of HER2 in PBS and incubated at 4 °C overnight. After washing, the plate was blocked using a blocking buffer (PBS containing 3% non-fat skim milk) at 37 °C for 1.5 h. Then, nanoparticles modified with 1F2 mAb through Mal-PEG5000-NHS and SPDP were added to the plate at different dilutions and kept for 1.5 h at 37 °C. Different concentrations of 1F2 mAb (1-100 ng/mL) were also added to obtain a standard curve. The washing step was repeated and HRP-conjugated sheep anti-mouse Ig was added and the plate was incubated for another 1.5 h at 37 °C. Following the final washing, the reaction was revealed with the TMB substrate. Finally, the reaction was terminated by 20% H_2_SO_4_ and the optical density (OD) measured by a multiscan ELISA reader (Organon Teknika, Turnhout, Belgium) at 450 nm. The amount of conjugated 1F2 to BSA nanoparticles with different crosslinkers was determined by the use of the standard curve. The study was carried out in triplicate and the results are reported as mean ± standard deviation (SD).

*Cellular binding assessment of nanoparticles by flow cytometry*

MCF7 and SKBR3 cells were stained indirectly at surface level. After harvesting the cells by trypsinization and washing with a washing buffer for two times (PBS, 0.1% BSA, 0.1% NaN_3_, pH 7.4), 10^6^ cells were incubated with 100 µL of 1F2-conjugated to 5-FU-loaded BSA nanoparticles (1 mg/mL) through Mal-PEG5000-NHS and SPDP at 4 °C for 1 hour. 1F2 mAb (2.5 µg/mL) and BSA nanoparticles were also involved as positive and negative controls, respectively. After incubation, the cells were washed twice with the washing buffer and then incubated with FITC conjugated sheep anti-mouse immunoglobulin at 4 °C for 1 hour. The cells were scanned with a flow cytometer (Partec, Nuremberg, Germany) after washing. The Flomax software (Partec) was used for data analyses.

*Physicochemical characterization of nanoparticle formulations* **.**

Particle size, polydispersity index (PDI) and zeta potential of BSA nanoparticles 5-FU-loaded BSA nanoparticles, 5-FU-loaded PEGylated BSA nanoparticles and 1F2-coupled 5-FU-loaded BSA nanoparticles were evaluated using a dynamic light scattering (DLS) apparatus (Malvern instrument Ltd., UK). The samples were diluted with distilled water before the analyses. In addition, the morphology of 1F2-coupled 5-FU-loaded BSA nanoparticles was analyzed using a scanning electron microscope (SEM) (KYKY-EM3200 model, China).

*In-vitro drug release study*

*In-vitro* cumulative release behavior of 5-FU from BSA nanoparticles, PEGylated BSA nanoparticles and 1F2-coupled BSA nanoparticles was evaluated during a period of 50 hours using dialysis method. The freeze-dried drug-loaded nanoparticle formulations with equal amount of 5-FU (1 mg) were suspended in separate dialysis tube bags and kept in 10 mL of PBS pH 7.4 at 37 °C in shaking water bath at 100 rpm. At predefined time intervals, PBS samples containing the released drug were taken and analyzed spectrophotometerically at 266 nm and then poured back into the release medium.

*Short and long-term storage stability investigation of mAb-modified drug-loaded nanoparticles*

The short time stability of the physicochemical properties and biological reactivity of 1F2-coupled 5-FU-loaded BSA nanoparticles were evaluated by DLS and ELISA during 72 hours of storage (time intervals of 1, 24, 48 and 72 hour after production) at 4 and 37 °C. In addition, the physicochemical and biological stability of 1F2-modified drug-loaded nanoparticles were assessed every two weeks during three months of storage at room temperature. In this regard, samples of nanoparticles containing 5% trehalose as preservative (17, 21) were freeze-dried, stored at room temperature and resuspended in distilled water before the analyses.

*In-vitro cytotoxicity evaluation*

*In-vitro* specificity and cytotoxicity effect of 1F2-coupled 5-FU-loaded BSA nanoparticles was evaluated on HER2-positive SKBR3 and compared with five other systems consisting of BSA nanoparticles, free 5-FU, 5-FU-loaded BSA nanoparticles, 5-FU-loaded PEGylated BSA nanoparticles and 1F2-coupled BSA nanoparticles. Briefly, cells (1 10^4^) were transferred into 96-well plates and incubated at 37 °C for 48 hours. After complete attachment of the cells, the supernatant was substituted with 100 µL of fresh media containing the mentioned systems with equal IC_30_ concentration of 5-FU (2 mM) (22) and nanoparticles (20 mg/mL). In addition, wells with no treatment were considered as control. In order to investigate the effect of contact time on cell specific attachment and cytotoxicity of the systems, cells were incubated with the nanoparticle formulations for 1 and 5 hours at 37 ^°^C. Our some pretests revealed that incubation time more than 5 hours did not increase the cytotoxicity of the systems and therefore, we considered 5 hours as the higher contact time. Then, the supernatant media were removed, fresh media was added to all wells and the cells were further incubated for 72 hours at 37 ^°^C. After the end of the incubation time, the cell viability was assessed by MTT assay. The medium was replaced by a mixture of fresh DMEM medium and MTT solution (5 mg/mL in PBS), followed by 2 hours incubation at 37 ^°^C. After dissolution of MTT with dimethylsulfoxide (DMSO, Sigma), the absorbance of the resulting solution was measured using a Microplate reader (Awareness Technology, USA) at a wavelength of 540 nm. The cell viability ratio was evaluated through comparing absorbance of treated cells against the untreated controls.

For control experiment, HER2 weakly expressing MCF7 cells were used. MCF7 cells were incubated with the systems at the same concentration of 5-FU and nanoparticles for 1 and 5 hours at 37 °C. After washing, the cells were further incubated for 72 hours at 37 °C. The cell viability assay was performed as described before.

## Results and Discussion

*Production of 1F2-coupled 5-FU-loaded BSA nanoparticles*

**Figure 1:**
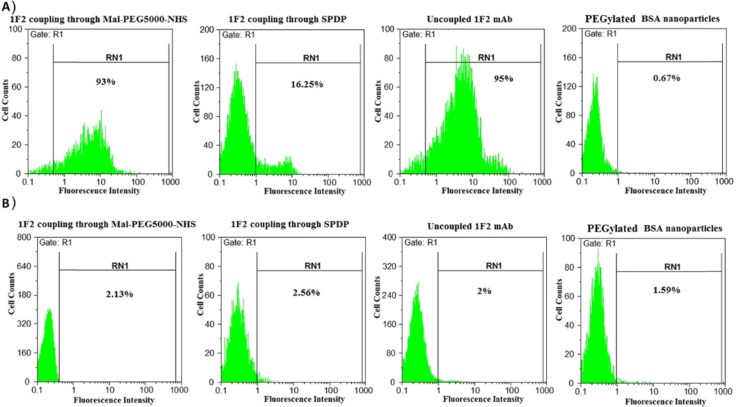
Cellular binding analyses using the flow cytometry technique. A) Cellular binding assessment of 1F2-coupled to 5-FU-loaded BSA nanoparticles through Mal-PEG5000-NHS and SPDP, PEGylated BSA nanoparticles (negative control) and uncoupled 1F2 mAb (positive control) on HER2-positive SKBR3 cells. B) Cellular binding assessment of 1F2-coupled to 5-FU-loaded BSA nanoparticles through Mal-PEG5000-NHS and SPDP, PEGylated BSA nanoparticles and uncoupled 1F2 mAb on HER2-negative MCF7 cells

BSA nanoparticles loaded with anticancer drug 5-FU were prepared and then, thiolated 1F2 mAb was covalently conjugated to the nanoparticles using two different heterobifunctional crosslinkers. ELISA results revealed 23 ± 4% and 8 ± 2% for conjugation efficiency of 1F2 mAb to 5-FU-loaded BSA nanoparticles by the use of Mal-PEG5000-NHS and SPDP, respectively, which demonstrates higher amount for Mal-PEG5000-NHS in comparison to SPDP. This amount was obtained for 1F2 coupling to unloaded HSA nanoparticles as reported previously (17). It means that the encapsulated therapeutics probably do not have serious effect on the conjugation of a monoclonal antibody as ligand to the surface of albumin based nanoparticles. In order to verify the efficacy of the produced targeted system, cellular binding of 1F2-coupled 5-FU-loaded BSA nanoparticles to SKBR3 and MCF7 cells was assessed. The flow cytometry results showed 93%, 16.25%, 95% and 0.67% of cellular binding on SKBR3 cells for 1F2 mAb conjugated to 5-FU-loaded BSA nanoparticles through Mal-PEG5000-NHS and SPDP, unconjugated 1F2 mAb (positive control) and BSA nanoparticles (negative control), respectively (Figure 1A). These systems showed no cellular binding on HER2-negative MCF7 cells (Figure 1B). Such a specific targeting for trastuzumab-modified HSA nanoparticles was also reported previously (23, 24). According to these findings, which are in accordance with our previous study (17), Mal-PEG5000-NHS was selected as the crosslinker between thiolated 1F2 mAb and 5-FU-loaded BSA nanoparticles and employed in the rest of this study.

*Characterization of nanoparticles*

**Figure 2:**
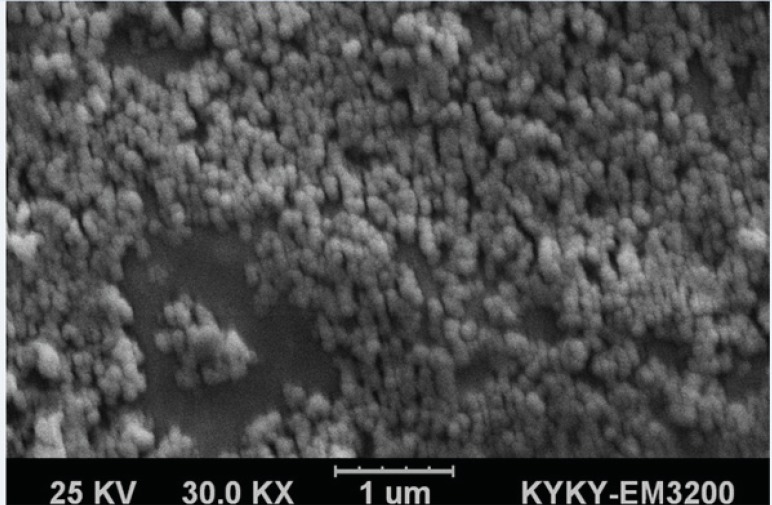
SEM micrograph of 1F2-coupled 5-FU-loaded BSA nanoparticles at magnification of 30000X

5-FU-loaded BSA nanoparticles were characterized in terms of particle size, PDI and zeta potential by DLS (Table 1). The average particle size of 1F2-conjugated 5- FU-loaded BSA nanoparticles was found to be 197 nm, which is higher than 5-FU-loaded BSA nanoparticles and approximately the same as PEGylated BSA nanoparticles. The mean PDI of 1F2-modified BSA nanoparticles is 0.051 that verifies their narrow size distribution. 1F2-modified BSA nanoparticles showed the surface charge of -22.5 mV. 1F2 mAb coupling did not significantly change the physicochemical properties of 5-FU-loaded BSA nanoparticles, which are in the proper range to be used in targeted drug delivery (17-21, 23-25). SEM analyses of 1F2-coupled 5-FU-loaded BSA nanoparticles verified their spherical morphology (Figure 2) as described previously (17, 25, 26).

**Table 1:** Physicochemical characterization of BSA nanoparticles, 5-FU-loaded BSA nanoparticles, 5-FU-loaded PEGylated BSA nanoparticles and 1F2-coupled 5-FU-loaded BSA nanoparticles (n=3, mean SD).

**Parameter**	**BSA nanoparticles**	**5-FU-loaded BSA nanoparticles**	**5-FU-loaded PEGylated nanoparticles**	**1F2-coupled 5-FU-loaded BSA nanoparticles**
Particle size (nm)	156±17	180±15	202±19	217±14
Polydispersity	0.050±0.013	0.049±0.01	0.050±0.017	0.051±0.02
Zeta potential (mV)	-31±4	-28.1±4.5	-20.5±3.6	-18.5±2.1

*In-vitro drug release study*

**Figure 3:**
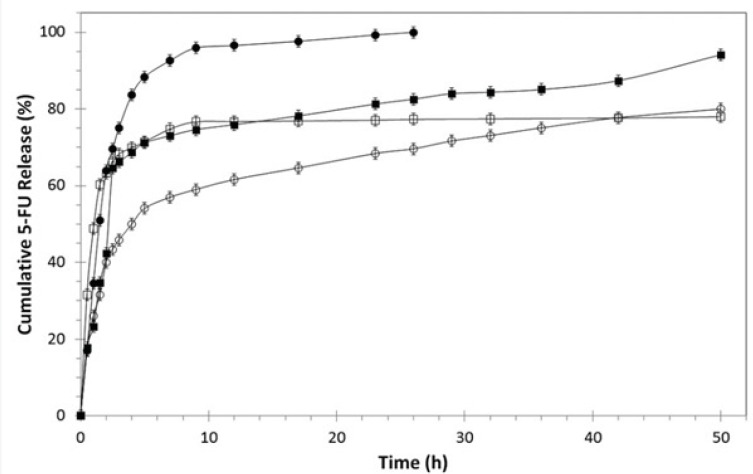
*In* *-* *vitro* cumulative release profiles of 5-FU from BSA nanoparticles (■), PEGylated BSA nanoparticles (□) and 1F2-conjugated BSA nanoparticles (○) in PBS (pH 7.4, 37 ^°^C) in comparison with the free drug (●) analyzed by dialysis method (n=3, mean SD).

Figure 3 represents the *in-vitro* cumulative release profiles of 5-FU from BSA nanoparticles, PEGylated and 1F2-conjugated BSA nanoparticle in PBS (pH 7.4, 37 °C) during 50 hours. All systems showed a two-phase release pattern consisting of an initial burst release followed by a slow sustained release stage. The initial burst effect could be related to the amount of the drug adsorbed on the surface of the nanoparticles. This initial burst release is slower for 1F2-coupled 5-FU-loaded BSA nanoparticles in comparison to free 5-FU and other systems, which can be associated with the presence of PEG and mAb and their role to hinder fast drug release. Subsequently, the entrapped drug in the inner core of the BSA nanoparticles diffuses slowly from the polymer matrix to the PBS medium and constitutes the slow 5-FU release phase.

*Storage stability of 1F2-coupled 5-FU-loaded BSA nanoparticles*

**Figure 4:**
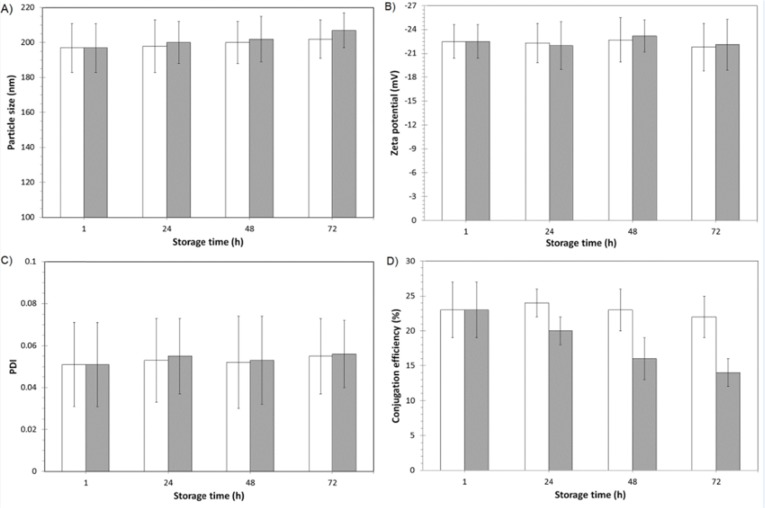
Short time storage stability of 1F2-coupled 5-FU-loaded BSA nanoparticles. A) Particle size (nm), B) zeta potential (mV), C) PDI and D) conjugation efficiency monitored during 72 h of storage at 4 (□) and 37 ^°^C (■) (n=3, mean SD).

The physicochemical properties of nanoparticles and biological reactivity of 1F2 mAb was analyzed by DLS and ELISA 1, 24, 48 and 72 hours after production and storage at 4 and 37 °C. DLS results showed that physicochemical properties of nanoparticles did not significantly change after 72 hours of storage at 4 and 37 °C (Figure 4). ELISA results demonstrated that the conjugation efficiency of 1F2-coupled 5-FU-loaded BSA nanoparticles were 23 4% and 22 5% after 1 and 72 hours of storage at 4 °C, respectively, which reveals preservation of the 1F2 biological reactivity in an appropriate level (Figure 4). In comparison to our previous study (17), the adsorbed and encapsulated drug in the matrix of albumin nanoparticles has no significant effect on biological reactivity of conjugated mAb. Nevertheless, the percentage of mAb conjugation decreased from 23 4% after 1 hour to 14 2% after 72 hours of storage at 37 °C, which indicates a substantial reduction of 1F2 biological reactivity. Therefore, short time storage of 1F2-coupled 5-FU-loaded BSA nanoparticles at 4 °C preserves the physicochemical properties of nanoparticles and biological reactivity of 1F2 mAb.

**Figure 5:**
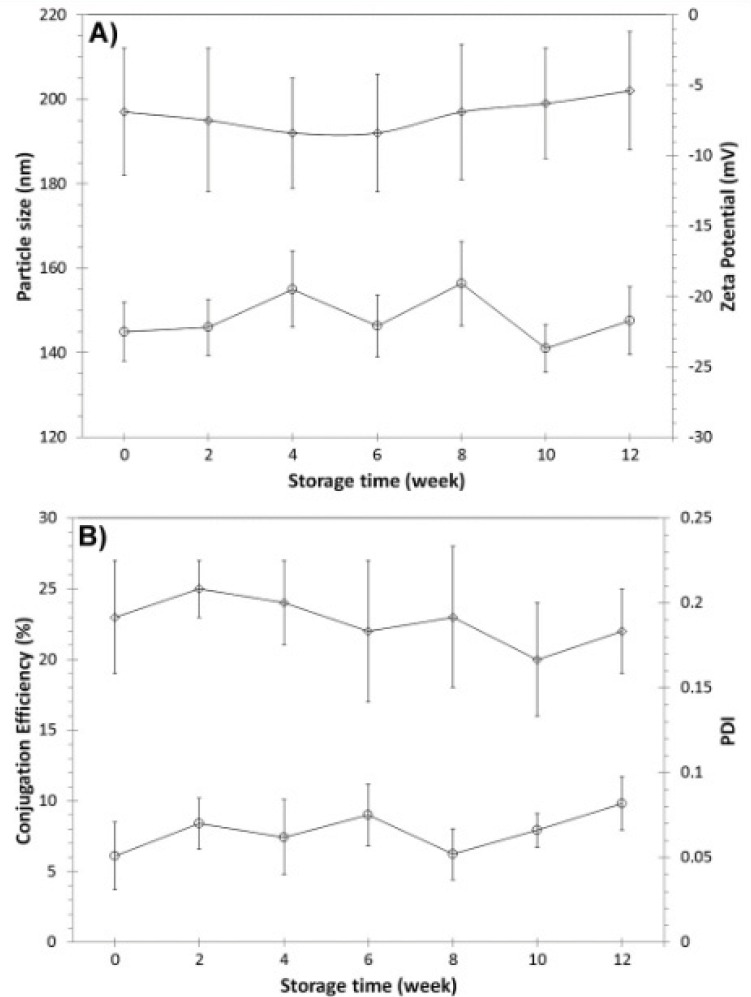
Long time storage stability of 1F2-coupled 5-FU-loaded BSA nanoparticles. A) Particle size (nm) ( ) and zeta potential (mV) ( ), B) conjugation efficiency ( ) and PDI ( ) recorded every two weeks during three months of storage in powder form at room temperature (n=3, mean SD).

The physicochemical properties and biological reactivity of targeted 5-FU delivery system was monitored during the longer period of three months of storage at room temperature. Nanoparticle powder was resuspended in distilled water before the analyses. Results showed no significant changes in physicochemical and biological properties of nanoparticles (Figure 5) after three months of storage at room temperature. These findings reveal that the powder form of this targeted drug delivery system maintains the physicochemical and biological properties of 1F2-conjugated 5-FU-loaded BSA nanoparticles.

*In-vitro cell viability*

**Figure 6:**
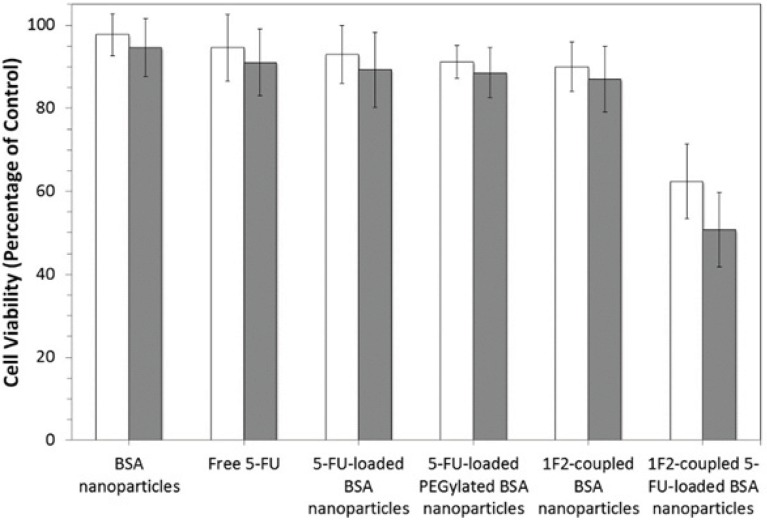
*In* *-* *vitro* cell viability assay. HER2-positive SKBR3 cells were incubated with six different systems including free 5-FU, BSA nanoparticles, 5-FU-loaded BSA nanoparticles, 5-FU-loaded PEGylated BSA nanoparticles, 1F2-copled BSA nanoparticles and 1F2-coupled 5-FU-loaded BSA nanoparticles for one (□) and 5 hours (■), then washed and incubated for another 72 hours in order to investigate their specificity and toxicity. Cells were assessed by MTT assay (n=3, mean SD).

Cytotoxicity of the BSA nanoparticles, free 5-FU, 5-FU-loaded BSA nanoparticles, 5-FU-loaded PEGylated nanoparticles and 1F2-coupled BSA nanoparticles was investigated *in-vitro* by MTT assay on SKBR3 cells and compared with the novel produced system. As is shown in Figure 6, the 1F2-coupled 5-FU-loaded BSA nanoparticles showed higher cell cytotoxicity (50.7 ± 9%) in comparison to other systems due to specific cell attachment through 1F2 mAb after washing, leading to accumulation of higher concentrations of 5-FU on targeted cells. Therefore, according to the results, the used concentration of drug (2 mM) is approximately equal to IC_50 _for 1F2-modified nanoparticles, which demonstrates higher toxicity of drug delivered by this targeted system. Other control systems such as 1F2-coupled nanoparticles did not show significant cytotoxicity, which is in accordance with previous studies (17, 23). Therefore, the most occurred cytotoxicity by drug-loaded modified-nanoparticles is due to their encapsulated 5-FU (37%) rather than 1F2 or nanoparticles. 5-FU toxicity more than 30% is probably due to higher rate of drug internalization by 1F2-modified nanoparticles. The higher 5-FU toxicity obtained by the targeted system in comparison to free drug (11 ± 8 %) after 5 hours contact with the cells continued with another 72 hours incubation, verifies that this system is more efficient.

The comparison of the cytotoxicity effect of this targeted 5-FU delivery system after 1 and 5 hours exposed to SKBR3 cells displayed higher cytotoxicity at longer incubation times due to longer cell attachment period leading to higher rate of nanoparticle internalization. Prolongation of the incubation time to more than 5 hours did not increase the cytotoxicity of 1F2-coupled 5-FU-loaded BSA nanoparticles.

To demonstrate the specificity of modified nanoparticles, MCF7 cells were incubated with all systems. None of the systems showed any significant cytotoxicity due to an unspecific uptake of the nanoparticle formulations. These findings verify the specificity and biological reactivity of 1F2-coupled 5-FU-loaded BSA nanoparticles. Our data demonstrate that this targeted system can be employed for efficient delivery of anticancer drug 5-FU to HER2-positive cancerous cells leading to improved therapeutic protocols.

## Conclusion

We demonstrated specific binding and intracellular accumulation of an anti-HER2 mAb-coupled BSA nanoparticles loaded with anticancer drug, 5-FU. A more effective 1F2 conjugation can be obtained by Mal-PEG5000-NHS as the crosslinker in comparison to SPDP. Employment of this targeted delivery system was able to enhance the therapeutic effect of 5-FU on HER2-positive cells. The combination of specific targeting with drug loading in these HSA based nanoparticulate system should lead to an improvement in cancer therapy. Further *in-vivo* analyses of this delivery system in an animal model will extend our understanding about its potential therapeutic efficacy and side effects.
